# Functional neuroanatomy of dopaminergic arousal systems: implications for the wake-promoting effect of psychostimulants, with particular reference to modafinil

**DOI:** 10.3389/fnana.2025.1670564

**Published:** 2025-12-09

**Authors:** Elemer Szabadi

**Affiliations:** Department of Psychiatry, University of Nottingham, Nottingham, United Kingdom

**Keywords:** arousal, dopaminergic nuclei, substantia nigra, VTA, nucleus accumbens, ventral pallidum, thalamus, psychostimulants

## Abstract

Arousal involves activation of the cerebral cortex by inputs from subcortical (hypothalamic, brainstem) wake-promoting nuclei, utilizing monoamine (noradrenaline, dopamine, serotonin, histamine) and neuropeptide (orexin) neurotransmitters. Dopaminergic neurones of the midbrain, originating from distinct nuclei [pars compacta of substantia nigra (SNc), ventral tegmental area (VTA), and other clusters of dopaminergic neurones in the ventral periaqueductal gray (vPAG)] and the pontine dorsal raphe nucleus (DRN), constitute a powerful wake-promoting system. Cortical activation by dopaminergic neurones can be due to either direct projections from the VTA and vPAG/DRN, to the cerebral cortex, or indirect projections from the VTA via the nucleus accumbens (NAc)/ventral pallidum (VP) and from the SNc via the thalamus. Stimulation of the VP, by inputs from the VTA via the NAc, can activate wake-promoting noradrenergic and orexinergic neurones, and stimulation of the thalamus, by inputs from the SNc, can activate wake-promoting glutamatergic thalamocortical neurones. There is also a two-way mutually reinforcing connection between the VTA/NAc/VP and SNc/thalamus systems, indicating the key role of the NAc in dopaminergic arousal regulation. Dopaminergic psychostimulants (e.g., amphetamine, cocaine) are highly addictive drugs of abuse, that activate both reinforcement mechanisms and promote wakefulness, by enhancing dopaminergic neurotransmission. The addictive potential of psychostimulants is related to the stimulation of reinforcement processes. Modafinil, an atypical psychostimulant, enhances wakefulness without affecting reinforcement, and thus is devoid of addictive potential. Unraveling the mode of action of modafinil may give insight into the neural mechanisms controlling reinforcement and arousal. Recent evidence indicates that the powerful arousal-enhancing effect of psychostimulants may mainly be due to indirect cortical activation via the NAc and thalamus.

## 1 Introduction

Psychostimulants are highly active addictive drugs of abuse. The best characterized drugs in this group are the dopaminergic psychostimulants, such as amphetamine and cocaine (for reviews, see McCreary et al., 2015; Djamshidian, 2015) The main effects of these drugs are the activation of reinforcement, a complex behavioral process underlying addiction (Koob, 1992; Nestler, 2005), and stimulation of some more simple behaviors, such as locomotion and arousal. While locomotor stimulation is prominent in experimental rodents (Gulley et al., 2004; Köks, 2015), promotion of wakefulness is the most striking effect in humans (Berridge and Arnsten, 2013; Trotti and Arnulf, 2021).

Psychostimulants have complex pharmacology. At the cellular level they interact with monoamine transporters that remove the released monoamine transporter from the synaptic gap into the presynaptic terminal (“uptake”) (Rothman and Baummann, 2003). Psychostimulants may have affinity for the transporters of all three monoamines (noradrenaline -NET, dopamine -DAT, serotonin -SERT), to varying degrees. For example, cocaine has approximately equal affinities for all three monoamine transporters, while amphetamine has high affinities for NET and DAT, and little affinity for SERT (Howell and Kimmel, 2008). Apart from monoamine transporters, psychostimulants may also interact with the vesicular transporter (VMAT2) that is responsible for the removal of a monoamine from the cytosol into synaptic vesicles. Amphetamine has a high affinity for VMAT2 leading to the release of dopamine from dopamine-containing tissues (Heal et al., 2013). The behavioral effects of psychostimulants (activation of reinforcement, stimulation of locomotion and arousal) are dose-dependent. This is illustrated by the dose-dependent dual effect of psychostimulants on cognitive function and locomotion: while a “low” dose has a stimulant effect, a “high” dose has an inhibitory effect (Berridge and Arnsten, 2013). Psychostimulants also have some important secondary effects, such as cognitive enhancement (“pro-cognitive effect”) (Wood et al., 2013; Spencer and Berridge, 2019) and appetite suppression (“anorectic effect”) (Corwin et al., 1987; Rothman and Baummann, 2003).

Psychostimulants have a propensity to addiction. Addiction develops as neuroplastic adaptation of the reinforcement circuit (mesolimbic pathway, see below) to repeated exposure to the drug. This adaptation has complex, and often contradictory, consequences: both reduced sensitivity (tolerance) and increased sensitivity (sensitisation) to the drug can develop. Tolerance may lead to negative emotional changes during drug withdrawal, and sensitisation may be responsible for drug craving and relapse (Nestler, 2005; Feltenstein et al., 2021).

The anatomical substrate of the actions of the psychostimulants on locomotion and reinforcement is the meso-striatal network. This network includes the striate nuclei (“striata”) of the basal ganglia, together with their inputs from the dopaminergic nuclei of the midbrain [pars compacta of the substantia nigra (SNc) and ventral tegmental area (VTA)]. The SNc projects to the dorsal striatum via the nigrostriatal pathway, and the VTA projects to the ventral striatum [also referred to as nucleus accumbens (NAc)] via the mesolimbic pathway. The meso-striatal network also includes the outputs from the striata via the associated pallidal nuclei (“pallida”). The network can be divided into a dorsal and a ventral section: dorsal striatum/dorsal pallidum (or globus pallidus) and ventral striatum (or nucleus accumbens/ventral pallidum) (Yelnick, 2002; Brodal, 2004; Ma, 2006; Lanciego et al., 2012). There is a functional division between the dorsal and ventral meso-striatal networks. The dorsal network is associated with locomotion (Muntean, 2020; Gatica et al., 2022) and modulation of cognition and sensorimotor behavior via the corticobasal ganglia/thalamic network (“cortico-striato-thalamic-cortical loop”) (Foster et al., 2021). The main function of the ventral network is regulation of reinforcement/motivation (Salamone and Correa, 2002; Chen, 2022). However, there is some overlap between the functions of the two networks: the dorsal striatum is also involved in reinforcement/motivation (Palmiter, 2008; Minogianis et al., 2019), and the ventral striatum (nucleus accumbens) can also play a role in locomotion (Dreher and Jackson, 1989; Essman et al., 1993).

In humans, apart from activation of reinforcement processes, the main effect of psychostimulants is promotion (“stimulation”) of arousal. Arousal can be defined both physiologically and behaviorally. The physiological definition of arousal is cortical activation as characterized by electroencephalography: (EEG): appearance of high-frequency/low-amplitude waves (“desynchronization”) (Lin et al., 2011). The behavioral definition of arousal is related to the three behavioral states (“vigilance states”): wakefulness, slow wave [or non-rapid-eye-movement (non-REM)] sleep, and REM sleep (Luppi and Fort, 2019; Gompf and Anaclet, 2020; Nir and de Lecea, 2023; Sulaman et al., 2023). Each vigilance state corresponds to a characteristic pattern of cortical activity: wakefulness and REM sleep - desynchronized (high frequency/low amplitude) EEG), non-REM sleep - synchronized (low frequency/large amplitude) EEG.

Arousal has two dimensions: vigilance and awareness (or “consciousness”). Vigilance reflects cortical activation, whereas awareness reflects mental activity. Vigilance and awareness are usually closely related. There is no awareness in the absence of vigilance (e.g., coma, anesthesia). No (or little) awareness may be present in states of considerable vigilance (e.g., vegetative state) (Boly et al., 2013). The vegetative state is a dissociated form of consciousness: the patient appears to be awake but is lacking any voluntary behavior. The vegetative state is contiguous with states of minimal consciousness (Laureys, 2005). The concept of arousal has been expanded by the inclusion of “attention,” as a third dimension, to complement vigilance and awareness (consciousness) (Oken et al., 2006; Pitts et al., 2018; Nani et al., 2019).

There is also a narrower definition of arousal: it is analogous to the vigilance state wakefulness (McGinley et al., 2015). This definition is based on the physiological definition of arousal: wakefulness is characterized by desynchronized EEG, presence of awareness and muscle tone (Luppi and Fort, 2019). Susceptibility to sensory stimulation is an important feature of wakefulness (Aston-Jones and Cohen, 2005; McGinley et al., 2015). In practical terms, “arousal” usually refers to a state of wakefulness, and “promotion of arousal” to a shift to the left on the “wakefulness – sedation – sleep” vigilance scale. This definition of arousal will be used in the subsequent discussion in relation to psychostimulants.

The term “alertness” is used to describe the degree of arousal (van Schie et al., 2021) There are a number of behavioral measures of alertness (e.g., critical flicker fusion frequency, power of pupil diameter fluctuations, self-rating scales of subjective alertness (for examples of the use of these measures, see Samuels et al., 2006, 2007; Hou et al., 2006).

The anatomical substrate of arousal is the neural network responsible for cortical activation. It has been known since the 1940s that a diffuse network of neurones in the reticular formation of the brainstem is crucial for cortical activation (Moruzzi and Magoun, 1949). This network is located subcortically in the diencephalon [thalamus (Gent et al., 2018; Ren J. et al., 2018) and hypothalamus (Szymusiak and McGinty, 2008; Haas and Lin, 2012; Yoshikawa et al., 2021)] and in the brainstem reticular formation (Steriade, 1996). The arousal-modulating reticular formation (“ascending reticular activating system”) extends into the basal forebrain (Miyawaki et al., 2023).

In the dispersed “sleep-arousal network’ it is possible to distinguish distinct wake-promoting and sleep-promoting nuclei (Szabadi, 2006, 2014, 2015b; Scammell et al., 2017; Gompf and Anaclet, 2020). Neurones in each type of nucleus use specific neurotransmitters: wake-promoting neurones use monoamines (noradrenaline, serotonin, dopamine, histamine), acetylcholine, glutamate, orexin, while sleep-promoting neurones use GABA, galanin, adenosine. There is a clear distinction between the projection targets of wake-promoting and sleep-promoting neurones: wake-promoting neurones project mostly directly to the cerebral cortex, whereas sleep-promoting neurones project mainly to wake-promoting nuclei. As discussed below (see section “2. Dopaminergic arousal systems”), dopaminergic arousal systems project indirectly to wake-promoting orexinergic neurones in the lateral hypothalamus and noradrenergic neurones in the locus coeruleus, located in the brain stem, and both these nuclei have bilateral connections with other nuclei, both wake-promoting and sleep-promoting, of the sleep-arousal network (Szabadi, 2015b).

The behavioral effects of psychostimulants (promotion of reinforcement, locomotion and wakefulness) reflect the activation of mesencephalic dopaminergic neurones. Dopaminergic activation is brought about by the inhibition of the dopamine transporter (DAT) responsible for removing released dopamine from the synaptic gap into dopaminergic nerve terminals (Rothman and Baummann, 2003; Wisor et al., 2001; Howell and Kimmel, 2008; dela Peña et al., 2015; Speranza et al., 2021.) It should be noted that psychostimulants inhibit not only DAT but also other monoamine transporters, such as NET and SERT (Rothman and Baummann, 2003; Howell and Kimmel, 2008), leading to an increase of the effects of both noradrenaline released from noradrenergic neurones in the locus coeruleus LC) and serotonin released from serotonergic neurones in the dorsal raphe nucleus (DRN). As both the LC and DRN are wake-promoting nuclei (Szabadi, 2015b), the blockade of NERT and SERT may contribute to the wake-promoting effect of psychostimulants. Interestingly, inhibition of NERT would also enhance the positive modulatory effect of noradrenaline on dopaminergic transmission in the prefrontal cortex (Masana et al., 2011; dela Peña et al., 2015).

There is evidence that the dopaminergic neurones of the midbrain constitute a component of the subcortical wake-promoting system (Monti and Monti, 2007; Monti and Jantos, 2008; Herrera-Solis et al., 2017; Wisor, 2019). The wake-promoting dopaminergic neurones are more dispersed than those responsible for promoting reinforcement and locomotion. Promotion of arousal involves cortical activation (Lin et al., 2011). Wake-promoting dopaminergic neurones may project directly to the cerebral cortex or may act indirectly by stimulating other cortically projecting wake-promoting neurones (e.g., noradrenergic, orexinergic, histaminergic, cholinergic, or glutamatergic neurones).

The effects of psychostimulants on arousal have been studied using neuroimaging and electrophysiology (EEG). Mainly two neuroimaging techniques have been used: positron emission tomography (PET) and functional magnetic resonance imaging (fMRI). It has been demonstrated with PET in humans that both amphetamine and methylphenidate release dopamine from dopamine containing nuclei, such as the dorsal and ventral striatum, substantia nigra, and some cortical regions (Martinez et al., 2003; Montgomery et al., 2007; for review, see Faraone, 2018). The atypical psychostimulant, modafinil, has also been shown to release dopamine from the dorsal and ventral striata, and to bind to DAT in both structures (Volkow et al., 2009) Using fMRI, the effects of psychostimulants on functional network connectivity have been studied. By simultaneously recording EEG and fMRI, level of arousal has been correlated with patterns of functional connectivity (Pourmotabbed et al., 2025). Modafinil, an effective wake-promoting psychostimulant, has been reported to alter the functional connectivity of thalamic nuclei with the neocortex (Pizzi et al., 2025), demonstrating the importance of the thalamocortical network in modafinil-induced arousal. Applications of EEG and neuroimaging have contributed to our understanding of the neurodevelopmental disorder ADHD (attention-deficit hyperactivity disorder) (Posner et al., 2020). EEG monitoring of arousal in patients with ADHD has revealed that ADHD is associated with low levels of arousal and unstable arousal regulation (Strauß et al., 2018). Psychostimulants are the treatment of choice for ADHD. It has been demonstrated with fMRI that the psychostimulant lisdexamfetamine alters striatal and thalamic connectivity differently in ADHD patients who show clinical improvement compared to unresponsive patients (Wang et al., 2022).

In the following sections, a review of the functional neuroanatomy of dopaminergic arousal systems will be presented, with special reference to the wake-promoting effect of psychostimulants.

## 2 Dopaminergic arousal systems

**FIGURE 1:**
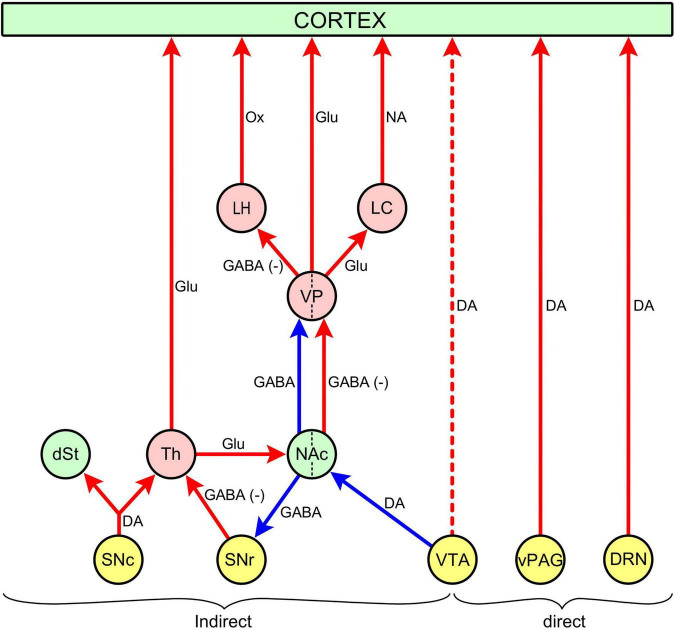
Direct and indirect projections form mesencephalic/pontine dopaminergic nuclei to the cerebral cortex. Nuclei (round disks); dopaminergic (yellow): DRN, dorsal raphe nucleus; vPAG, ventral periaqueductal gray; VTA, ventral tegmental area; SNc, substantia nigra, pars compacta; SNr, substantia nigra, pars reticulata; primary projection targets (green): NAc, nucleus accumbens; dSt, dorsal striatum; secondary and tertiary projection targets (pink): Th, thalamus; VP, ventral pallidum; LH, lateral hypothalamic area; LC, locus coeruleus, Connections (arrows: red - excitatory/ stimulatory; blue - inhibitory. Continuous red arrows to cortex: wake-promotion; broken red arrow to cortex: promotion of cognitive function (for details, see section “2.1.1.5. Promotion of cognitive function by the VTA”). Neurotransmitters: DA, dopamine, GABA, γ-aminobutyric acid, GABA(-), inhibited GABAergic connection leading to disinhibition (“excitation”) of target, Glu, glutamate, Ox, orexin, NA, noradrenaline.

Figure 1 is a schematic diagram of the cortical projections of dopaminergic arousal systems Dopaminergic neuronal clusters of the midbrain promote wakefulness by activating the cerebral cortex, either directly or indirectly. The directly acting systems include the VTA in the ventral tegmentum, projecting to the cerebral cortex via the mesocortical pathway, and the vPAG and DRN in the dorsal mesencephalic tegmentum/pons, directly innervating cortical neurones. The indirectly acting systems include the VTA, projecting to the cerebral cortex via the NAc/VP (“mesolimbic system”), and the SNc, projecting to the cortex via the thalamus (“nigrothalamic system”).

### 2.1 Directly acting systems

#### 2.1.1 Ventral tegmental area (VTA)/mesocortical system

##### 2.1.1.1 Cellular composition and connections of the VTA

The VTA contains the group of dopaminergic neurones labeled A10 in the classification of catecholaminergic neurones of the brainstem (Dahlström and Fuxe, 1964; Nieuwenhuys, 1985; Oades and Halliday, 1987; Björklund and Dunnett, 2007). Apart from dopaminergic neurones, the VTA also contains GABAergic and glutamatergic neurones (Morales and Margolis, 2017). These neurones function as both interneurons, that modulate the activity of dopaminergic neurones, and projection neurones synapsing with neurones in other structures. Some of the dopaminergic neurones contain either GABA or glutamate as co-transmitters (Trudeau et al., 2014; Apergis-Schoute et al., 2019).

Using an array of modern neuroscientific techniques, such as electron microscopy (Briggman and Bock, 2012), electrophysiology (Kim et al., 2017), viral vectors (Chen et al., 2019), optogenetics and chemogenetics (Vlasov et al., 2018), both the afferents to the VTA, and the efferents from the VTA have been mapped out in detail. Furthermore, in many instances, it has been possible to identify the type of neurone (dopaminergic, GABAergic or glutamatergic) receiving input from another structure or sending output to a distant target. The VTA receives inputs from the prefrontal cortex (PFC), the NAc, pedunculopontine and laterodorsal tegmentum, periaqueductal gray (PAG), lateral hypothalamus, ventral pallidum (VP), dorsal raphe nucleus (DRN). There are outputs from the VTA to the PFC, NAc, amygdala, hippocampus, VP, periaqueductal gray (PAG), bed nucleus of the stria terminalis, olfactory tubercle, locus coeruleus (LC; Morales and Margolis, 2017). The best studied outputs are those to the PFC, NAc, and amygdala (Walsh and Han, 2014).

##### 2.1.1.2 Mesocortical pathway

There is a well-established direct connection from the VTA to the PFC (Oades and Halliday, 1987; Domesick, 1988; Van den Heuvel and Pasterkamp, 2008). Furthermore, VTA neurones projecting to the PFC form a distinct cluster in the medial-posterior part of the VTA (Lammel et al., 2012), and have some distinct characteristics (Walsh and Han, 2014). Optogenetic stimulation of dopaminergic neurones in the VTA has been reported to excite both principal and parvalbumin-positive interneurons in the PFC. It has been suggested that the co-release of glutamate with dopamine may contribute to the excitatory response to VTA stimulation (Pèrez-López et al., 2018; Zhong et al., 2020). Furthermore, it has been proposed that the release of glutamate from dopamine/glutamate neurones onto pyramidal neurones and interneurons would produce a temporally more distinct signal than the diffuse modulation by dopamine alone (Lapish et al., 2007).

##### 2.1.1.3 Cortical control of VTA function

A unique feature of the VTA is that its activity is controlled by afferents from its projection target in the PFC. Interestingly, two other cortically projecting subcortical nuclei, the laterodorsal tegmental nucleus and the median raphe nucleus, share this feature (Souza et al., 2022). It is well documented that there is a glutamatergic projection, originating from the pyramidal neurones of the PFC, to the VTA (Carr and Sesack, 2000; Sesack et al., 2003; Wu et al., 2013; Souza et al., 2022). It has been shown that the projection from the PFC to the VTA shows some target specificity: dopaminergic neurones in the VTA receiving an input from the PFC give rise to mesocortical outputs, while GABAergic neurones targeted from the PFC give rise to mesoaccumbens outputs (Carr and Sesack, 2000).

##### 2.1.1.4 Wake-promotion by the VTA

Until relatively recently there has been no convincing information about the potential role of the VTA in modulating vigilance states. However, during the past decade, this situation was changed with the application of genetics-based neuroscience techniques, such as optogenetics and chemogenetics (English and Roth, 2015; Vlasov et al., 2018), to stimulate identified VTA neurones.

It has been reported in mice that both optogenetic (Eban-Rothschild et al., 2016) and chemogenetic (Oishi et al., 2017a) activation of dopaminergic neurones in the VTA increase arousal. Glutamatergic and GABAergic neurones also contribute to the modulation of vigilance states by the VTA. Chemogenetic activation of glutamatergic neurones has been reported to evoke wakefulness and REM sleep, whereas activation of GABAergic neurones to induce NREM (Slow-Wave) sleep (Yu et al., 2019). The modulation of vigilance states by the VTA is confirmed by another study using the pharmacogenetic “designer receptors exclusively activated by designer drugs (DREADD)” approach. Activation of VTA neurones expressing the excitatory muscarinic designer receptor hM3Dq promoted arousal, whereas activation of VTA neurones expressing the inhibitory muscarinic designer receptor hM4Di promoted sleep (Sun et al., 2017).

The functional significance of the role of the VTA in promoting arousal is highlighted by the observation that optogenetic activation of VTA dopamine neurones induces reanimation from general anesthesia (Taylor et al., 2016). For reviews of the role of the VTA in arousal, see Oishi and Lazarus, 2017; Wisor, 2019.

Interestingly, it has been shown that optogenetic stimulation of axon terminals of dopaminergic neurones projecting from the VTA to the NAc increases arousal, whereas stimulation of dopaminergic axon terminals from the VTA to the prefrontal cortex is without effect (Eban-Rothschild et al., 2016). Therefore, activation of VTA neurones is likely to lead to cortical activation indirectly via the mesolimbic pathway rather than directly via the mesocortical pathway. The wake-promoting effect of the mesolimbic output from the VTA is confirmed by the observation that chemogenetic stimulation of the mesolimbic projection decreased sleep and reduced cortical EEG power, in contrast to the effect of stimulation of the nigrostriatal projection that had the opposite effect (Qiu et al., 2019). It has been shown that that the dopaminergic output from the VTA to the NAc may promote arousal by stimulating D2 dopamine receptors since the arousal-enhancing effect of VTA activation is blocked by the D2/D3 antagonist raclopride (Oishi et al., 2017a). It is plausible that the D2 receptors are located on neurones in the NAc. In conclusion, the VTA promotes arousal mainly indirectly via the NAc [for details, see section “2.2.1. Mesolimbic system (NAc/VP)”].

##### 2.1.1.5 Promotion of cognitive function by the VTA

The direct projection from the VTA to the prefrontal cortex via the mesocortical pathway is responsible mainly for promoting executive cognitive functions controlled by the prefrontal cortex (Floresco and Magyar, 2006; Jenni et al., 2017; Ohtake et al., 2024). Interestingly, cognitive enhancement (“procognitve effect”) is an important corollary of arousal-enhancement by psychostimulants (Wood et al., 2013; Spencer and Berridge, 2019). The procognitive effect of psychostimulants is likely to reflect potentiation of the activating effect of the mesocortical pathway on the prefrontal cortex (Peterson et al., 1990; Nesbit et al., 2024). The importance of the mesocortical pathway in controlling cognitive functions is highlighted by the observation that cognitive impairment in cerebral small vessels disease (SVD) was related to damage to the mesocortical pathway. Interestingly, other key features of SVD, apathy and gait disturbance, also appeared to be associated with damage to the mesocortical pathway (Li et al., 2024).

#### 2.1.2 Dorsal mesencephalic/pontine system (vPAG/DRN)

##### 2.1.2.1 Ventral periaqueductal gray (vPAG)

A cluster of dopaminergic neurones has been identified in the vPAG that are active during wakefulness and quiescent during sleep, and whose destruction prolongs the sleep period (Lu et al., 2006). These neurones project to the prefrontal cortex where they evoke an activating effect by stimulating both D1 and D2 dopamine receptors. Both receptor types are present in the cerebral cortex (Richfield et al., 1989; Lidow et al., 1991), where both D1 receptors (Lewis and O’Donnell, 2000) and D2 receptors (Robinson and Sohal, 2017) may evoke excitation of cortical neurones.

Apart from projecting to the prefrontal cortex, wake-promoting dopaminergic neurones in the vPAG also have bi-directional connections to both wake-promoting (cholinergic, noradrenergic, serotonergic, orexinergic) and sleep-promoting (GABAergic) neurones of the sleep-arousal network (Lu et al., 2006).

The noradrenergic projection from the LC to the dopaminergic neurones of the vPAG has been studied in detail (Porter-Stransky et al., 2019; Petersen et al., 2025) Activation of this projection, like chemogenetic activation of dopaminergic neurones in the vPAG, promotes arousal. This effect seems to be mediated via α1-adrenoceptors located on astrocytes stimulating a glutamatergic input to dopaminergic neurones in the vPAG (Porter-Stransky et al., 2019). As activation of dopaminergic neurones in the vPAG by stimulation of α1-adrenoceptors could be prevented by pharmacological blockade of adenosine A_2A_ (excitatory) receptors, it was suggested that adenosine A_2A_ receptors may also play a role in linking astrocytic adrenergic signaling to wake-promoting dopaminergic neurones (Petersen et al., 2025).

The dopaminergic neurones of the vPAG, like those of the VTA (Zhou et al., 2015), have been implicated in anesthesia (Li et al., 2018; Liu C. et al., 2020), and may play a role in the recovery from anesthesia (Wang J. et al., 2023). The wake-promoting role of the vPAG dopaminergic neurones is highlighted by clinical reports of severe excessive daytime sleepiness in neurodegenerative Lewy body diseases, such as multiple system atrophy and dementia with Lewy bodies, leading to the loss of these neurones (Benarroch et al., 2009).

##### 2.1.2.2 Dorsal raphe nucleus (DRN)

Although the DRN is mainly a serotonergic nucleus (Ren J. et al., 2018), it also contains a sparse cluster of dopaminergic neurones that are contiguous with the dopaminergic neurones of the vPAG (Lu et al., 2006; Cho et al., 2017). In fact, the dopaminergic neurones in the vPAG and DRN constitute a unified dopaminergic system. It has become customary to refer to the two nuclei as a combined unit: “periaqueductal gray/dorsal raphe” (for examples, see Yu et al., 2021; Zhao et al., 2022). The definition of DRN^DA^ used by Cho et al. (2017) incorporates all dopaminergic neurones in the dorsal midline pontine raphe nucleus (DRN) and the dorsal mesencephalic vPAG.

Cho et al. (2017) expanded the findings of Lu et al. (2006) in the vPAG using modern genetics-based neuroscience techniques (optogenetics, chemogenetics) in the DRN^DA^. The activity of DRN^DA^ neurones fluctuated in the course of the sleep/wakefulness period, highest activity occurring during wakefulness. These neurones could be activated by arousal-evoking salient stimuli. Optogenetic activation of DRN^DA^ neurones promoted wakefulness, and chemogenetic inhibition opposed wakefulness.

In conclusion, the dopaminergic neurones of the vPAG/DRN constitute an effective dedicated arousal system that directly modulates the activity of the prefrontal cortex. Moreover, the vPAG/DRN is closely integrated within the sleep/arousal network. In contrast to the dopaminergic neurones of the ventral mesencephalic tegmentum, these neurones do not modulate other functions, such as locomotion (SNc) or reinforcement (NAc). Indeed, it has been shown that DRN dopaminergic neurones, in contrast to DRN serotonergic and VTA dopaminergic neurones, fail to reinforce operant responding (McDevitt et al., 2014).

### 2.2 Indirectly acting systems

#### 2.2.1 Mesolimbic system (NAc/VP)

##### 2.2.1.1 Cellular composition

###### 2.2.1.1.1 Nucleus accumbens (ventral striatum)

The nucleus accumbens has the same cytoarchitecture as the dorsal striatum (DSt; Yelnick, 2002; Ma, 2006; Marcott et al., 2018). Approximately 96% of the cells are GABAergic medium spiny neurones (MSNs). The MSNs are the projection neurones from the striata. The striata also contain a number of cholinergic and GABAergic interneurons. The MSNs of the NAc, like those in the DSt, can be divided into two groups according to their dopaminergic input: D1-MSNs and D2-MSNs. D1-MSNs are excited by dopamine via D1 dopamine receptors, whereas D2-MSNs are inhibited by dopamine via D2 dopamine receptors (Surmeier et al., 2007, 2011). In the NAc, MSNs also carry adenosine receptors: D1-MSNs are associated with A_1_R (inhibitory) adenosine receptors, and D2-MSNs are associated with A_2A_R (excitatory) adenosine receptors (Oishi et al., 2017b; Oishi and Lazarus, 2017). In the DSt, D1- and D2- MSNs are segregated according to their projection targets: D1-MSNs are linked to the “direct pathway” projecting directly to the substantia nigra, pars reticulata (SNr) and internal segment of the globus pallidus (GPi), whereas D2-MSNs reach these targets indirectly via the external segment of the globus pallidus (GPe; Gerfen and Surmeier, 2011). Although it is possible to distinguish between direct and indirect output pathways also in the NAc, there is no clear segregation of D1- and D2-MSNs between these pathways: both types of MSN can send outputs via both the direct and indirect pathways (Kupchik et al., 2015; Kupchik and Kalivas, 2017).

###### 2.2.1.1.2 Ventral pallidum

The **ventral pallidum** is the principal output structure from the NAc. Due to its ventral location, it is also classified as part of the basal forebrain. Apart from being a relay station between the NAc and the ventral mesencephalon (VTA, SN), and a large number of other structures lying outside the NAc/VP circuit, such as the forebrain, brainstem, and limbic structures, it also has extensive roles in processing reinforcement (Root et al., 2015; Kupchik and Prasad, 2021; Soares-Cunha and Heinsbroek, 2023). There are three types of projection neurones in the VP: GABAergic (inhibitory), glutamatergic (excitatory), cholinergic (excitatory). The majority of output neurones are GABAergic, and project to most output targets. Many targets receive both GABAergic and glutamatergic projections (Kupchik and Prasad, 2021; Soares-Cunha and Heinsbroek, 2023).

##### 2.2.1.2 Connections

The main **input to the NAc** is from the VTA (Sesack and Grace, 2010: Volkow et al., 2017; see also section “2.1.1. Ventral tegmental area (VTA)/mesocortical system”). The NAc also receives inputs from the prefrontal cortex (Montaron et al., 1996), paraventricular thalamus (Perez and Lodge, 2018), and hippocampus (Perez and Lodge, 2018).

The outputs from the NAc arise from D1- and D2-MSNs. D1-MSNs innervate the ventral mesencephalon (SNc, SNr, VTA) (striato-mesencephalic pathway), whereas both D1- and D2-MSNs innervate the VP (striato-pallidal pathway) (Pardo-Garcia et al., 2019). The NAc is closely associated with the VP, its main output structure.

**FIGURE 2:**
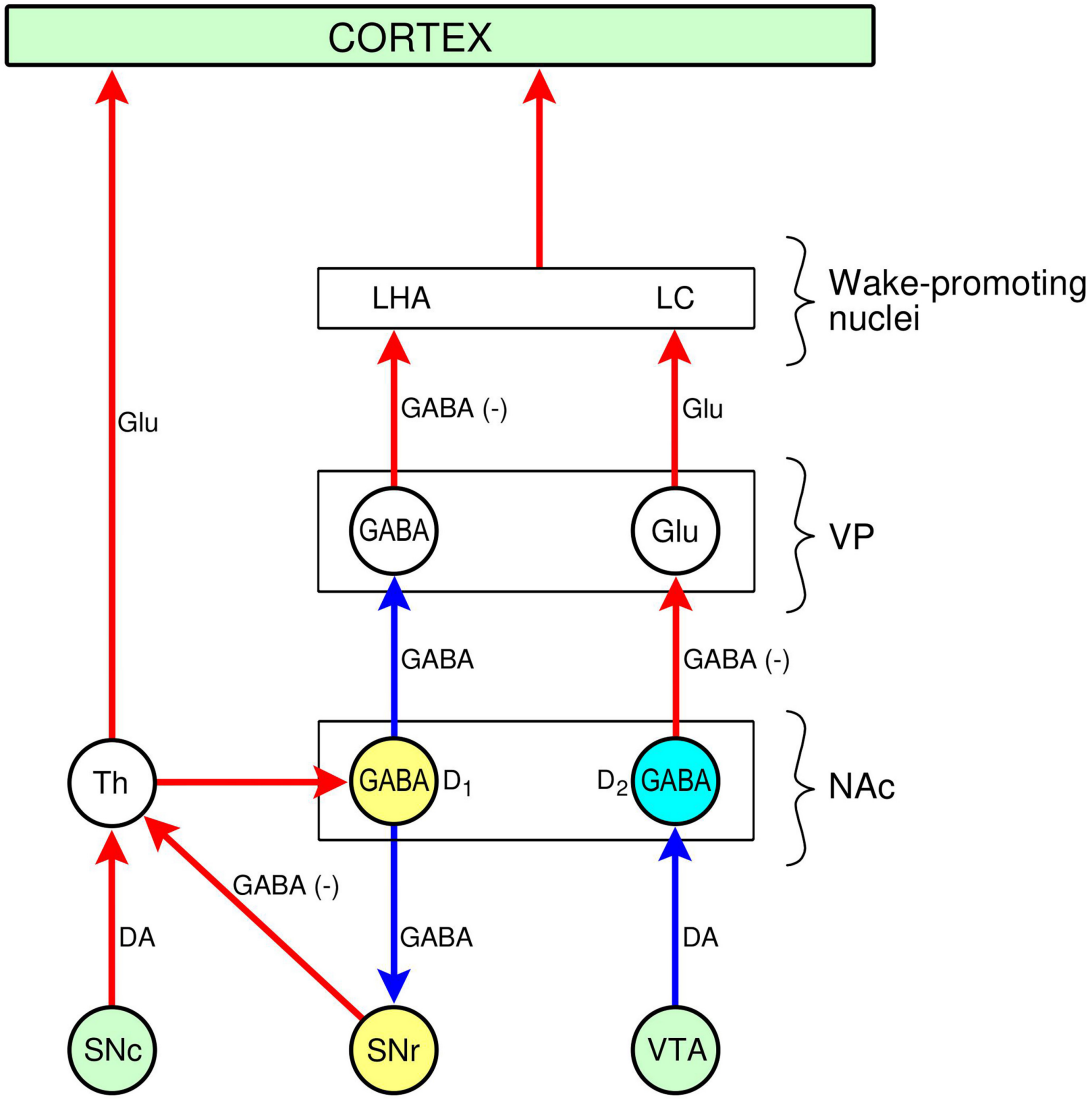
Projections from the nucleus accumbens (NAc) to the cerebral cortex via the ventral pallidum (VP) and the thalamus. Nuclei (round disk): VTA, ventral tegmental area; SNc, substantia nigra, pars compacta; SNr, substantia nigra, pars reticulata; Th, thalamus; D2-GABA, D2-NSNs (medium spiny. neurones); GABA-D1, D1-MSNs; Glu, glutamate; GABA, γ-aminobutyuric acid; LHA, lateral hypothalamic area; LC, locus coeruleus. Connections: arrows, red: excitatory; blue: inhibitory. Neurotransmitters: DA: dopamine, GABA, γ-aminobutyric acid, GABA(-), inhibited GABAergic connection leading to disinhibition (“excitation”) of target, Glu: glutamate.

The striato-pallidal pathway projects to GABAergic and glutamatergic output neurones in the VP that link the NAc to a vast array of structures outside the immediate projection area of D1- and D2-MSNs (Figure 2). These more remote targets include the prefrontal cortex, habenula, subthalamic nucleus, lateral hypothalamus, pons/medulla oblongata (DRN, LC, pedunculopontine nucleus), mesencephalon (SNc, SNr, VTA, NAc, vPAG), and amygdala (Haber et al., 1985; Zahm, 1989; Groenewegen et al., 1993; for review, see Soares-Cunha and Heinsbroek, 2023). The “remote targets” contain a number of wake-promoting areas (prefrontal cortex, DRN, vPAG, LC, LHA, pedunculopontine nucleus) whose activation plays an important role in mediating the wake-promoting effect of the NAc.

The projection from D1-MSNs to the VP appears to play a role in reinforcement/motivation processes since it has been shown to be required for cue-induced cocaine seeking (Pardo-Garcia et al., 2019).

##### 2.2.1.3 Modulation of vigilance states

The NAc/VP plays an important role in modulating sleep and arousal. The GABAergic neurones of D1- and D2-MSNs in the NAc, projecting to the VP, seem to play opposite roles: D1-MSNs promote wakefulness, whereas D2-MSNs promote sleep (Oishi and Lazarus, 2017; Wisor, 2019). The anatomical basis for this dichotomy lies in the NAc/VP circuitry (see Figure 2).

###### 2.2.1.3.1 Role of D1-MSNs

Stimulation of D1-MSNs, projecting to GABAergic output neurones in the VP, would inhibit them, leading to disinhibition of their target neurones and thus activation of the wake-promoting nuclei LC and LHA). GABAergic neurones in the VP have been shown to promote arousal by projecting to both orexinergic neurones in the LHA and dopaminergic neurones in the VTA (Li et al., 2021).

The role of D1-MSNs in promoting arousal has been demonstrated experimentally (Luo et al., 2018). It was shown with fiber photometry that the population activity of D1-MSNs displays arousal-related increases. Optogenetic activation of D1-MSNs induced transition from NREMS (Non-REM-Sleep) to wakefulness, and chemogenetic stimulation evoked prolonged wakefulness. The wake-promoting effect of D1-MSNs was ascribed to their projections to mesencephalic (dopaminergic, GABAergic, glutamatergic) neurones, and hypothalamic orexinergic neurons in the LHA.

###### 2.2.1.3.2 Role of D2-MSNs

Stimulation of D2-MSNs, projecting to glutamatergic output neurones in the VP, would inhibit them, leading to the de-activation of the wake-promoting nuclei (LC, LHA) targeted by them, and thus facilitating the development of sleep. In agreement with this model, it has been shown that optogenetic stimulation of glutamatergic neurones in the VP increases wakefulness (Luo et al., 2023).

The role of D2-MSNs in promoting sleep has been studied experimentally. Optogenetic activation of D2-MSNs in the NAc induced Slow-Wave (Non-REM) Sleep, whereas chemogenetic inhibition of the same neurones suppressed Slow-Wave Sleep [(Oishi et al., unpublished observations, cited by Oishi and Lazarus (2017)]. These observations were confirmed in a detailed study in NAc neurones expressing excitatory A_2A_ adenosine receptors. These neurones correspond to D2-MSNs (see section “2.2.1.1. Cellular composition, above). Optogenetic or chemogenetic activation of these neurones induced Slow-Wave Sleep, and chemogenetic inhibition of the same neurones prevented the induction of sleep (Oishi et al., 2017b).

Inhibition of D2-MSNs by dopamine, released from a dopaminergic input, is expected to evoke a wake-promoting effect. Indeed, it has been shown that optogenetic and chemogenetic stimulation of dopaminergic neurones in the VTA activates dopaminergic nerve terminals reaching the NAc, accompanied by an increase in arousal (Eban-Rothschild et al., 2016; Oishi and Lazarus, 2017) (see section “2.1.1.4. Wake-promotion by the VTA”). The dopaminergic input from the VTA to the NAc would stimulate inhibitory D2 dopamine receptors on D2-MSNs projecting to excitatory glutamatergic neurones in the VP. Inhibition of the D2-MSNs would disinhibit the glutamatergic neurones projecting to extra-VP wake-promoting neurones, leading to an increase in arousal (see Figure 2). The wake-promoting effect of inhibition of the indirect pathway to the VP, originating from the D2-MSNs, is in agreement with the observation that this pathway can also be inhibited by motivational stimuli leading to suppression of sleep (Oishi et al., 2017b).

#### 2.2.2 Nigrothalamic systems (SN/thalamus)

The role of the thalamus in controlling sleep and arousal is well recognized (Schiff, 2008; Gent et al., 2018). Mesencephalic dopaminergic neurones may influence thalamic neuronal activity via two pathways: the nigrothalamic pathway, projecting from the SNc to central thalamic nuclei, and the nucleus accumbens/nigrothalamic circuit, projecting from D1-MSNs in the NAc or from the SNc, via the DSt, to the dorsomedial thalamic nucleus via the GABAergic neurones of SNr.

##### 2.2.2.1 Nigrothalamic pathway (SNc/midline thalamus)

The nigrothalamic pathway originates from a small contingent of dopaminergic neurones in the most ventral part of the SNc intruding into the SNr (Cebrián and Prensa, 2010). Some of these project to the thalamus, others to the striatum, and some of the striatum-projecting cells also send collaterals to the thalamus (Freeman et al., 2001). Dopaminergic terminals have been described to reach midline and intralaminar thalamic nuclei, known to be involved in mediating arousal via widespread “non-specific” or “higher order” cortical projections (Groenewegen and Berendse, 1994; Van der Werf et al., 2002; Saalmann, 2014; Vertes et al., 2022). The midline nuclei include the paraventricular nucleus (“paraventricular thalamus,” PVT), the focus of much recent research aimed at clarifying the cellular mechanisms and neuronal circuitry involved in thalamic arousal (Kolaj et al., 2014; Ren S. et al., 2018; Shao et al., 2019; Ao et al., 2021; Bu et al., 2022a,b; Wang Y.-L. et al., 2023).

The activity of excitatory glutamatergic projection neurones of the PVT is modulated by opposing GABAergic (inhibitory) and dopaminergic (excitatory) inputs. GABAergic afferents to the PVT, from the suprachiasmatic and arcuate nuclei of the hypothalamus (Kolaj et al., 2014), inhibit the glutamatergic neurones via GABA_*A*_ receptors. The dopaminergic input from the SNc increases the activity of glutamatergic neurones via D2 dopamine receptors (Ao et al., 2021). Two mechanisms have been proposed for this effect: a direct one and an indirect one. Dopamine may stimulate excitatory D2 dopamine receptors (Robinson and Sohal, 2017) on the cell bodies of the glutamatergic neurones (Ao et al., 2021). Alternatively, dopamine may stimulate release-inhibiting heteroreceptors on GABAergic nerve terminals (Floran et al., 1997) innervating the glutamatergic neurones, thereby disinhibiting them (Beas et al., 2018).

The glutamatergic neurones of the PVT change their activity in line with the level of consciousness during anesthesia (Wang Y.-L. et al., 2023) and may play a role in recovery from anesthesia (Bu et al., 2022a). Stimulation of D2 dopamine receptors in the PVT by the D2 receptor agonist quinpirole has been shown to shorten the time required for emergence from anesthesia (Ao et al., 2021), while the D2 dopamine receptor antagonist raclopride delays the time required for recovery from anesthesia.

The wake-promoting effect of the PVT has been attributed to an excitatory glutamatergic projection to the NAc (Ren J. et al., 2018; Shao et al., 2019) where it stimulates D1-MSNs (Luo et al., 2018). The GABAergic D1-MSNs, projecting to GABAergic output neurones in the VP, would inhibit them, leading to the disinhibition of wake-promoting orexinergic neurones in the LHA and noradrenergic neurones in the LC innervated by the GABAergic output from the VP (Figure 2; see also section “2.2.1.3. Modulation of vigilance states”). Interestingly, the LHA and LC project back to the PVT (Ren J. et al., 2018, Shao et al., 2019), thereby creating a self-reinforcing arousal circuit: PVT → NAc → VP → LHA/LC → PVT.

There are clinical implications of the wake-promoting effect of the SNc/thalamus arousal system. In Parkinson’s disease, degeneration of dopaminergic neurones in the SNc leads to the loss of the dopaminergic output to the thalamus (Freeman et al., 2001). The resultant reduction in thalamic arousal may contribute to the excessive daytime sleepiness of patients suffering from Parkinson’s disease (Arnulf, 2005). Furthermore, hypersomnia has been reported as a consistent symptom in paramedian thalamic stroke (Bassetti et al., 1996; Herman et al., 2008), probably reflecting the loss of the wake-promoting function of the PVT. However, apart from the loss of dopaminergic neurones in the SNc, the loss of wake-promoting dopaminergic neurones in other nuclei, like the VTA and vPAG, may also contribute to excessive daytime sleepiness in Parkinson’s disease and related neurodegenerative disorders (e.g., Lewy body dementia) (Szabadi, 2024).

##### 2.2.2.2 Nucleus accumbens/nigrothalamic circuit (NAc/mediodorsal thalamus)

The NAc projects to the SN both directly from D1-MSNs and indirectly from D2-MSNs via the VP (Pardo-Garcia et al., 2019; Figures 1, 2). This GABAergic projection innervates the GABAergic neurones of the pars reticulata (SNr). The SNr is a major output target of D1-MSNs: it sends equal projections to the SNr and the VP (Attachaipanich et al., 2023). The SNr projects to the medio-dorsal nucleus of the thalamus that innervates the prefrontal cortex (Montaron et al., 1996) via the glutamatergic thalamocortical pathway (Lambe et al., 2003) Electrical and chemical stimulation of the NAc have been shown to inhibit the spontaneous discharge of the GABAergic neurones in the SNr (Deniau et al., 1994). Therefore, it would be expected that the output from the NAc to the SNr may promote arousal by disinhibiting the glutamatergic excitatory thalamocortical output to the prefrontal cortex.

There is a self-reinforcing circuit between the NAc and the prefrontal cortex. The NAc projects to the prefrontal cortex via the SNr and mediodorsal thalamus, and the prefrontal cortex projects back to the NAc (Montaron et al., 1996).

The SNr is also a key node in the corticobasal ganglia-thalamic network (“cortico-striato-thalamic-cortical loop”) (Foster et al., 2021). Glutamatergic neurones in the cerebral cortex project to GABAergic neurones of the D1- and D2-MSNs in the DSt. The D1-DMSNs project to the SNr via the direct pathway (“striatonigral pathway”), whereas the D2-MSNs project to the external segment of the globus pallidus (GPe) (striatopallidal pathway), that in turn is connected to the SNr. The SNr is the main output structure of the DSt, modulating the activity of corticothalamic and locomotor circuits (Kreitzer and Malenka, 2008; Freeze et al., 2013) The SNr projects to the parafascicular and ventromedial nuclei nuclei of the thalamus that in turn send outputs to the cerebral cortex (Foster et al., 2021).

Apart from arousal [see above, and Liu D. et al., 2020], the SNr has also been implicated in motor control (Liu D. et al., 2020; Attachaipanich et al., 2023) and reinforcement behavior (Attachaipanich et al., 2023). Motor control may be mediated by the projection of the SNr to the “mesencephalic locomotor region” (MLR; Fougére et al., 2019; Dautan et al., 2021), and reinforcement behavior by an output from the SNr to the NAc via the “SNr →’ mediodorsal thalamus → prefrontal cortex → NAc” circuit (Montaron et al., 1996).

There is evidence of a connection from the NAc to the SNc. It has been reported that high frequency stimulation of the NAc suppressed the spontaneous firing of dopaminergic neurones in the SNc (Sesia et al., 2014). Through this connection the NAc may modulate the outputs from the SNc to the thalamus both via the nigrothalamic pathway [see section “2.2.2.1. Nigrothalamic pathway (SNc/thalamus)”] and the nigrostriatal pathway, projecting to the thalamus indirectly through the “DSt → globus pallidus → SNr → ventromedial thalamus” route (Foster et al., 2021).

## 3 Modafinil: a psychostimulant with relative selectivity for arousal mechanisms

Psychostimulants have two main effects in humans: promotion of reinforcement and enhancement of arousal. Promotion of reinforcement can lead to addiction and drug abuse, and is therefore deleterious, whereas enhancement of arousal can be clinically useful, and is therefore desirable. Most psychostimulants affect reinforcement and arousal in approximately equal measure (Berridge and Arnsten, 2013), significantly limiting their clinical use. Modafinil is an atypical psychostimulant: it is an effective wake-promoting drug with little or no potential for abuse (Hersey and Tanda, 2024). Therefore, modafinil has replaced typical psychostimulants, such as amphetamine or methylphenidate, in the treatment of clinical conditions when raising the level of arousal is desirable (e.g., narcolepsy and ADHD) (Hersey et al., 2021; Hersey and Tanda, 2024). Due to its selective and potent wake-promoting effect, modafinil has also been used as a research tool to explore arousal mechanisms (e.g., Wisor et al., 2001; Qu et al., 2008; Wisor, 2013; Yang et al., 2021).

### 3.1 Mechanism of wake-promotion

#### 3.1.1 Interaction with dopamine transporter (DAT)

Like typical psychostimulants (e.g., amphetamine, cocaine) modafinil binds to the dopamine transporter (DAT; Mignot et al., 1994; Loland et al., 2012; for review, see Wisor, 2019). Blockade of DAT by modafinil has also been demonstrated in the human brain *in vivo* using positron emission tomography (PET; Volkow et al., 2009). Inhibition of DAT underlies the wake-promoting effect of modafinil. Indeed, in DAT knockout mice modafinil fails to enhance arousal (Wisor et al., 2001). The blockade of dopamine uptake leads to the enhancement of extracellular concentration of dopamine in the striatum, a structure rich in dopaminergic terminals (Wisor et al., 2001; Murillo-Rodriguez et al., 2007; Zolkowska et al., 2009). Modafinil, however, may bind to DAT in an atypical way, leading to its atypical behavioral profile (see section “3.2. Potential mechanism of relative selectivity for arousal”).

#### 3.1.2 Interaction with dopamine receptors

Apart from inhibition of the dopamine transporter, dopamine receptors also seem to be necessary for the arousal-enhancing effect of modafinil. It was reported that the combined application of D1 and D2 dopamine receptor antagonists could prevent the wake-promoting effect of modafinil, similarly to the application of a D1 receptor antagonist in D2 receptor knockout mice (Qu et al., 2008).

#### 3.1.3 Action on striatum

The neurochemical and physiological effects of modafinil have been studied in the striatum, a structure rich in dopaminergic terminals. The blockade of dopamine uptake by modafinil leads to the enhancement of extracellular concentration of dopamine in the striata (Wisor et al., 2001; Murillo-Rodriguez et al., 2007; Zolkowska et al., 2009. Furthermore, modafinil activates phasic dopamine signaling, characteristic of typical psychostimulants, in dose-dependent manner, in dorsal and ventral striata (Bobak et al., 2016). There is accumulating evidence that the wake-promoting effect of modafinil may be mediated by the striata, in particular the ventral striatum (NAc). It has been reported that lesioning the core of the NAc abolished the induction of arousal by modafinil in rats, while lesioning the shell of the NAc did not influence the effect of modafinil (Qiu et al., 2012).

A detailed study in mice investigated the role of the striata in mediating modafinil-induced wakefulness (Yang et al., 2021). Dopaminergic neurones, in the VTA and SNc, identified by the presence of DAT, were lesioned. In the control animals, modafinil increased wakefulness and caused accumulation of dopamine, in a dose-dependent manner, in both the NAc and DSt, projection targets of dopaminergic neurones in the VTA and SNc, respectively. Lesioning DA neurones in the VTA substantially reduced the effect of modafinil, whereas lesioning DA neurones in the SNc slightly reduced it. Finally, lesioning DA neurones in both the NAc and DSt completely abolished the effect of modafinil. On the other hand, lesioning dopaminergic neurones in the DRN did not affect wake-promotion by modafinil. It was concluded that mesencephalic dopaminergic neurones were essential for modafinil-induced arousal.

The findings of Yang et al. (2021) are consistent with mediation of modafinil’s arousal-enhancing effect by the mesolimbic and nigrothalamic wake-promoting systems. The mesolimbic dopaminergic neurones in the VTA project to the ventral striatum (NAc) [see section “2.2.1. Mesolimbic system (NAc/VP)”], and the wake-promoting function of this pathway is well established (see section “2.1.1.4. Wake-promotion by the VTA”). Lesioning of dopaminergic neurones in the VTA substantially reduced the wake-promoting effect of modafinil (Yang et al., 2021). This finding is in agreement with the report of Qiu et al. (2012) who found that lesioning the core of the NAc abolished the induction of arousal by modafinil.

The nigrothalamic dopaminergic neurones in the SNc project to the thalamus, either independently or as collaterals of nigrostriatal neurones [see section “2.2.2.1. Nigrothalamic pathway (SNc/thalamus)”]. Modafinil increases locomotor activity in rodents (Simon et al., 1996), consistent with stimulation of nigrostriatal neurones. Lesioning dopaminergic neurones in the SNc caused a modest decrease in modafinil-evoked arousal (Yang et al., 2021), suggesting the attenuation of modafinil-evoked arousal via the nigrothalamic arousal system. Modafinil may also promote thalamic arousal via the nigrostriatal pathway: modulating the activity of MSNs in the DSt could lead to stimulation of the SNr via the globus pallidus, and the SNr is known to project to wake-promoting thalamocortical neurones [see section “2.2.2.2. Nucleus accumbens/nigrothalamic circuit (NAc/mediodorsal thalamus)”].

In contrast to the activation of mesolimbic and nigrothalamic neurones, modafinil does not seem to have any effect on the vPAG/DRN arousal-modulating dopaminergic system [see section “2.1.2. Dorsal mesencephalic/pontine system (vPAG/DRN)”]. The report of Yang et al. (2021) suggests that the arousal-enhancing effect of modafinil is largely mediated via the circuitry of the NAc/VP system [see section “2.2.1. Mesolimbic system (NAc/VP)”].

#### 3.1.4 Activation of orexinergic neurones (LHA)

It has been shown that modafinil increases the release of histamine, a wake-promoting neurotransmitter, from the tuberomamillary nucleus (TMN) in the hypothalamus. Furthermore, the stimulation of histamine release by modafinil is absent in orexinergic neurone-deficient mice, suggesting that the activation of histaminergic neurones by modafinil is triggered by orexinergic neuronal activity (Ishizuka et al., 2010). However, it is unlikely that activation of orexinergic neurones is the mechanism responsible for the therapeutic effect of modafinil in narcolepsy, since there is a loss of orexinergic neurones in this sleep disorder (Mahoney et al., 2019).

#### 3.1.5 Activation of noradrenergic neurones (LC)

The central noradrenergic system plays a major role in mediating the wake-promoting effect of psychostimulants in general, and of modafinil in particular (Berridge, 2006; España et al., 2016).

The LC is a major wake-promoting nucleus, occupying a central position in the sleep/arousal network. It activates the cerebral cortex both directly and indirectly. The direct cortical activation is via a diffuse network of efferents stimulating excitatory α_1_-adrenoceptors on cortical neurones (Szabadi, 2013), whereas the indirect activation via the NAc involves both excitatory outputs, via α_1_-adrenoceptors, to other wake-promoting nuclei (e.g., LHA, TMN), and an inhibitory output, via α_2_-adrenoceptors, to the sleep-promoting ventrolateral preoptic nucleus (VLPO) of the hypothalamus (Szabadi, 2015b) Modulation of arousal by the LC is closely associated with the modulation of autonomic activity, increasing arousal being accompanied by increasing sympathetic activity (for reviews, see Samuels and Szabadi, 2008; Szabadi, 2013, 2018).

The LC receives a dopaminergic input from the VTA. There is evidence of both a direct (“mesocoerulear pathway”) and an indirect connection from the VTA to the LC. Dopaminergic fibers reach the LC from the VTA (Deutch et al., 1986; Ornstein et al., 1987; Maeda et al., 1994), and both D1 and D2 dopamine receptors are present in the LC (Mansour and Watson, 1995). Furthermore, stimulation of the VTA with kainic acid was reported to lead to the activation of the prefrontal cortex via the LC (Deutch et al., 1986). However, VTA stimulation may also activate the LC via an indirect route, through the NAc/VP. The VP projects to the LC via glutamatergic and GABAergic neurones, and either activation of glutamatergic neurones, or disinhibition of GABAergic neurones can lead to the activation of the LC (see section “2.2.1.3. Modulation of vigilance states”; Figure 2).

Modafinil has no direct effect on noradrenergic neurones: it does not interact with adrenoceptors and has very low affinity for NET (Hersey and Tanda, 2024). However, some *in vivo* studies produced results consistent with inhibition of NET by modafinil. This observation was attributed to an indirect effect of modafinil via the inhibition of DAT (Madras et al., 2006).

Modafinil may increase the activation of noradrenergic neurones in the LC via the indirect route (VTA → NAc → VP → LC). Although both D1- and D2-MSNs in the nucleus accumbens may be involved in LC activation, only D2-MSNS may be stimulated by inhibition of DAT in NAc-projecting VTA dopaminergic neurones, since on dopaminergic neurones projecting to D1-MSNs modafinil may act as an atypical DAT inhibitor (see section “3.2. Potential mechanism of relative selectivity for arousal”).

The activity of noradrenergic neurones in the LC is regulated by recurrent collaterals stimulating inhibitory α_2_-adrenoceptors on the cell body and dendrites (“somatodendritic autoreceptors”). These autoreceptors act to dampen the firing rate of the neurones (Huang et al., 2012; Szabadi, 2015a). The α_2_-adrenoceptor agonist clonidine has sedative and sympatholytic effects, that are likely to reflect the de-activation (“switching off”) of the LC (Szabadi and Bradshaw, 1996). It was reported in human volunteers that single doses of clonidine and modafinil had opposite effects on the measures of alertness and sympathetic activity, clonidine reducing and modafinil increasing the measures (Hou et al., 2005). The contrasting effects of clonidine and modafinil on arousal and sympathetic activity, functions modulated by the LC, are consistent with contrasting effects of the two drugs on the LC, clonidine de-activating and modafinil activating the noradrenergic nucleus.

It has been proposed that the LC may operate in two modes, a more general tonic mode and a more specific phasic mode leading to stimulation of selective targets. Target specific phasic activation is driven by the outcome of task-related decision processes (Aston-Jones and Cohen, 2005). It has been shown in humans with functional MRI that the activation of the LC by modafinil leads to a shift in the relationship between tonic and phasic activity: tonic activity decreasing and phasic activity increasing. The increase in task-related LC activity leads to increased task-related activity of the prefrontal cortex (PFC), confirming the role of the LC in PFC function and suggesting a mechanism for the procognitive effect of modafinil (Minzenberg et al., 2008).

### 3.2 Potential mechanism for relative selectivity for arousal

Psychostimulants promote both arousal and reinforcement by interacting with the NAc/VP dopaminergic system. Modafinil differs from other psychostimulants: it preferentially enhances arousal with relatively little effect on reinforcement processes (Vosburg et al., 2010; Mereu et al., 2017; Yepez and Juárez, 2022) and thus is devoid of abuse/addiction potential (Hersey et al., 2021; Hersey and Tanda, 2024). Therefore, it has been labeled “an atypical stimulant” (Hersey and Tanda, 2024).

As motivation is closely related to reinforcement (Volkow et al., 2017; Johnson et al., 2022), modafinil is not expected to have any effect on motivation. However, contrary to this prediction, there are reports that modafinil can promote motivated behavior (Young and Geyer, 2010; Camargos and Quintas, 2011; Ecevitoglu et al., 2024). It has been argued that arousal, apart from enhancing motivated behavior mediated by reinforcement, may also promote reinforcement *per se* (Berridge and Arnsten, 2013; España et al., 2016; Kaźmierczak and Nicola, 2022). This suggestion is in agreement with a recently proposed theoretical framework according to which there are two forms of motivation: “extrinsic,” driven by reinforcers, and “intrinsic,” independent of reinforcement processes (Karayanni and Nelken, 2022; Morris et al., 2022). Therefore, arousal may act as an “intrinsic” motivator, and modafinil may increase motivation by virtue of its wake-promoting activity.

The pharmacology of modafinil has been investigated in detail (Minzenberg and Carter, 2008; Mereu et al., 2013; Hersey et al., 2021; Hersey and Tanda, 2024). The effects of modafinil have been studied on different neurotransmitter systems. The most prominent effect of modafinil is on the dopaminergic system: like other dopaminergic psychostimulants it increases dopaminergic neurotransmission by inhibiting DAT [see section “3.1.1. Interaction with dopamine transporter (DAT)”]. Modafinil has little affinity for other neurotransmitter transporters and has no affinity for neurotransmitter receptors. However, it alters the levels of neurotransmitters in several brain regions. Apart from the level of dopamine, modafinil increases the levels of noradrenaline, orexin, serotonin, histamine and glutamate, and decreases the level of GABA These effects, however, may be indirect, reflecting the activation of different neurotransmitter systems via the VTA/NAc/VP circuit (see section “2.2.1.2. Connections”; Figure 2).

The molecular pharmacology of modafinil’s binding to DAT shows some peculiarities. When psychostimulants bind to DAT, they alter the conformation of the DAT protein in a characteristic way. Typical psychostimulants, like cocaine, promote an open, outward facing conformation, while the binding of modafinil to DAT promotes a closed, inward-facing conformation. Therefore, modafinil has been classified as an “atypical DAT inhibitor” (Loland et al., 2008; Schmitt and Reith, 2011).

Recent developments of the molecular pharmacology of DAT inhibitors, may be relevant for understanding the atypical behavioral profile of modafinil. A number of “atypical DAT inhibitors” have been identified (Schmitt et al., 2013; Reith et al., 2015; Hong et al., 2018), and some important patterns have emerged. It has been shown that molecular atypicality of the DAT inhibitor was associated with behavioral atypicality of the psychostimulant concerned, such as failure to promote reinforcement/motivation and absence of abuse potential. Atypical DAT inhibitors are being developed as potential treatments for psychostimulant abuse (Zhang et al., 2017; Soler-Cedeno et al., 2025).

Modafinil’s lack of effect on reinforcement /motivation and abuse potential may also be related to the atypicality of its interaction with DAT. However, while atypical DAT inhibition may deprive modafinil of an effect on reinforcement/motivation, the drug still can lead to the promotion of arousal, another dopamine-mediated function (see section “3.1.3. Action on striatum”). Therefore, it has to be assumed that reinforcement/motivation and arousal are likely to be mediated by separate neuronal circuits within the NAc/VP system, and that the dopaminergic outputs from the VTA to distinct reinforcement and arousal circuits would be affected differently by modafinil.

It has been shown that cue-induced cocaine seeking, indicating operation of reinforcement processes, was mediated by the projection of D1-MSNs from the NAc to the VP, whereas the projection from D2-MSNs to the VP was not involved (Pardo-Garcia et al., 2019). This observation suggests that only the projection from D1-MSNs may mediate reinforcement, while outputs from both D1- and D2-MSNs may be involved in mediating arousal (see section “2.2.1.3. Modulation of vigilance states”) Therefore, modafinil’s relative selectivity for arousal may be due to the preferential activation of the dopaminergic input to D2-MSNs rather than D1-MSNs. However, it should be noted that there is some controversy regarding the role of D2-MSNs in mediating reinforcement: no role (Pardo-Garcia et al., 2019), stimulation and inhibition (Olivetti et al., 2020) have all been reported.

The molecular basis of the selectivity of modafinil for arousal may be related to the way DAT operates in dopaminergic neurones projecting to D1- and D2-MSNs. Modafinil may behave as an atypical DAT inhibitor of D1-MSNs-projecting dopaminergic neurones, while it may behave as a typical DAT inhibitor of D2-MSNs-projecting dopaminergic neurones. This implies that there are differences between D1-MSNs-projecting and D2-MSNs-projecting neurones. Dopaminergic neurones in the VTA do not form a unified population, and there may be different contingents with different characteristics (Serra et al., 2021). The heterogeneity of dopaminergic neurones may reflect the operation of different forms of DAT (e.g., oligomers) in some contingents of dopaminergic neurones (Das et al., 2019; Jayaraman et al., 2021). Therefore, there is a possibility that dopaminergic neurones with different characteristics may project to D1- and D2-MSNs. It would be necessary to confirm this hypothesis experimentally.

The dopaminergic neurones of vPAG/DRN would be a good candidate for mediating the arousal-enhancing effect of modafinil since these neurones do not activate reinforcement processes [see section “2.1.2.2. Dorsal raphe nucleus (DRN)”]. However, it has been reported that lesioning the DRN fails to disrupt the wake-promoting effect of modafinil (Yang et al., 2021).

## 4 Interaction of dopaminergic drugs with dopaminergic arousal systems

Due to the complexity of the dopaminergic arousal systems, dopaminergic drugs may affect their components differentially. It has been reported that the D2/D3 dopamine receptor agonist pramipexole, while it enhances locomotion and reinforcement, consistent with activation of dopaminergic neurones in the SNc and VTA, reduces the level of arousal, indicating de-activation of dopaminergic arousal systems (Szabadi, 2024). A possible reason for the paradoxical effect of pramipexole on arousal may lie in the differential effect of the drug on different components of the dopaminergic arousal systems. While pramipexole may enhance arousal by stimulating postsynaptic D2 dopamine receptors on D2-MSNs in the NAc, it may also reduce arousal by stimulating presynaptic release-inhibiting D2 receptors in the vPAG. If the effect on the vPAG predominates it may supersede the effect on the NAc, resulting in sedation. Interestingly, the effect of pramipexole on the pupil reveals both effects of pramipexole: the pupil is dilated while alertness is reduced. This is a paradoxical finding since reduction in alertness is usually associated with pupil constriction. The pupil dilation evoked by pramipexole may reflect activation of the LC via the NAc/VP system, while the reduction in alertness may reflect the switching-off of the vPAG system (Szabadi, 2024).

## 5 Therapeutic uses of psychostimulants

The therapeutic uses of psychostimulants are related to their wake-promoting action, and to some other actions closely related to wake-promotion. There are a number of disorders that may benefit from treatment with psychostimulants.

### 5.1 Disorders of arousal

Deficit in arousal can have serious psychopathological consequences. Hypersomnia (“excessive daytime sleepiness”) (Gandhi et al., 2021; Trotti and Arnulf, 2021) and ADHD (Strauß et al., 2018; Strauß et al., 2020) constitute important categories of arousal disorders. Causes of hypersomnia include inadequate sleep, sleep-disordered breathing, circadian rhythm sleep-wake disorders, and central disorders (narcolepsy, idiopathic hypersomnia). Narcolepsy is a severe complex sleep disorder caused by the loss of orexinergic neurones in the lateral hypothalamus. Hypersomnia is only one of its features (Bassetti et al., 2019; Mahoney et al., 2019). ADHD is a neurodevelopmental disorder (Posner et al., 2020). that is associated with low levels of arousal and unstable arousal regulation (Strauß et al., 2018; Strauß et al., 2020). Psychostimulants are the main lines of treatment both for narcolepsy and ADHD.

### 5.2 Cognitive impairment

Psychostimulants have a facilitatory effect on cognitive processes (“cognitive enhancement,” “procognitive effect”) (Wood et al., 2013; Spencer and Berridge, 2019). The anatomical basis of this effect is a dedicated output from the VTA to the prefrontal cortex (mesocortical pathway) (see section “2.1.1.2. Mesocortical pathway”; Figure 1). The effects of psychostimulants on two cognitive functions, working memory and sustained attention, are well established (Spencer and Berridge, 2019). Amphetamine and diphenhydramine used to be used for cognitive enhancement (Wood et al., 2013), however, they have been largely superseded by modafinil (Minzenberg and Carter, 2008; Mereu et al., 2013).

### 5.3 Fatigue in multiple sclerosis

Fatigue is a common distressing feature of multiple sclerosis (Ramirez et al., 2021). The mechanisms underlying fatigue in multiple sclerosis are not fully understood (Russo et al., 2017; Manjaly et al., 2019; the possible involvement of dopaminergic mechanisms has been suggested (Dobryakova et al., 2015). Psychostimulants have been used, with variable results, in an attempt to relieve it (Nourbakhsh et al., 2021; Natsheh et al., 2021). A deficit of arousal may also be associated with fatigue in multiple sclerosis that may be relieved with modafinil (Niepel et al., 2013).

### 5.4 Neurorehabilitation

Psychostimulants are also used to facilitate the rehabilitation of patients with complex neurological injuries following traumatic brain injury and stroke (Kraus, 1995; Whyte et al., 2002).

## 6 Conclusion

The physiological basis of arousal is activation of the cerebral cortex by subcortical structures. The two major dopaminergic nuclei of the midbrain (SN, VTA), together with the dopaminergic neurones of the vPAG/DRN, contribute to arousal by sending excitatory projections to the cortex. While other dopaminergic functions, like locomotion and reinforcement, are modulated by well-defined unitary networks (locomotion: SNc/DSt; reinforcement: VTA/NAc/VP), arousal is modulated by a more dispersed system, involving all three dopaminergic neurone clusters. In fact, there are several dopaminergic arousal systems, projecting to the cerebral cortex either directly or indirectly via other wake-promoting nuclei (Figure 1). There are direct cortical outputs from the VTA, vPAG and DRN (a pontine extension of the dopaminergic neurones of vPAG). Of these only the outputs from vPAG/DRN have a wake-promoting role, while the output from the VTA to the prefrontal cortex (mesocortical pathway) mainly stimulates executive cognitive functions. There are indirect cortical outputs from the NAc/VP and SNc/thalamus. The VP projects to other wake-promoting nuclei, such as the LC and LHA, thereby fully integrating the dopaminergic arousal systems with the sleep/arousal network. There are two-way connections between the NAc/VP and SN/thalamus system, integrating the functions of the two systems. By being integrated with both the sleep/arousal network and the SN/thalamus system, the NAc/VP occupies a key position in dopaminergic arousal regulation.

The dopaminergic psychostimulants (e.g., amphetamine, cocaine) block the dopamine transporter (DAT), and the resultant inhibition of dopamine uptake into the nerve terminals increases dopaminergic neurotransmission, leading to activation of the postsynaptic cells in the DSt, NAc, thalamus, and cerebral cortex. Through this mechanism, psychostimulants act as potent activators of locomotion, reinforcement and arousal, functions controlled by the mesencephalic dopaminergic neurones. Of these functions, reinforcement and arousal are prevalent in humans, enhancement of reinforcement/motivation being responsible for the addiction/abuse potential of these drugs, whereas promotion of wakefulness is the basis of their therapeutic potential.

Modafinil is a relatively arousal-selective psychostimulant with little effect on reinforcement and therefore with little or no abuse potential. This suggests that, although reinforcement and arousal may be both mediated via the NAc/VP system, they may act via different pathways. It would be of interest to identify these pathways experimentally (see section “3. Modafinil: a psychostimulant with relative selectivity for arousal mechanisms”).

Due to the complexity of the distribution of dopamine receptors, both presynaptic and postsynaptic, within the wake-promoting dopaminergic systems, it is difficult to predict the overall effect on arousal of a dopamine receptor agonist. An example is the D2/D3 receptor agonist pramipexole, used in the treatment of Parkinson’s disease, that contrary to the prediction of an alerting effect causes sedation. This could be interpreted as the effect of the drug on the vPAG → cerebral cortex system superseding its effect on the VTA → NAc → VP → LC →cerebral cortex and VTA → NAc thalamus → cerebral cortex systems (see section “4. Interaction of dopaminergic drugs with dopaminergic arousal systems”),

The psychostimulants have important clinical uses. Due to their arousal-enhancing effect they are useful in the treatment of arousal disorders, such as hypersomnia (“excessive daytime sleepiness”) and ADHD. Their cognitive enhancing (“nootropic”) effect can be helpful for the treatment of cognitive disorders. They are also used to treat fatigue conditions, such as fatigue complicating multiple sclerosis. Diphenhydramine and amphetamine are the psychostimulants used traditionally in clinical situations; however, they are being increasingly superseded by modafinil (see section “5. Therapeutic uses of psychostimulants”).
